# Use of intensity quotients and differences in absolute structure refinement

**DOI:** 10.1107/S2052519213010014

**Published:** 2013-05-17

**Authors:** Simon Parsons, Howard D. Flack, Trixie Wagner

**Affiliations:** a EaStCHEM School of Chemistry and Centre for Science at Extreme Conditions, The University of Edinburgh, King’s Buildings, West Mains Road, Edinburgh EH9 3JJ, Scotland; b Chimie minérale, analytique et appliquée, University of Geneva, Geneva, Switzerland; c Novartis Institutes for BioMedical Research, 4002 Basel, Switzerland

**Keywords:** intensity quotients, absolute structure refinement

## Abstract

Several methods for absolute structure refinement were tested using single-crystal X-ray diffraction data collected using Cu *K*α radiation for 23 crystals with no element heavier than oxygen: conventional refinement using an inversion twin model, estimation using intensity quotients in *SHELXL*2012, estimation using Bayesian methods in *PLATON*, estimation using restraints consisting of numerical intensity differences in *CRYSTALS* and estimation using differences and quotients in *TOPAS-Academic* where both quantities were coded in terms of other structural parameters and implemented as restraints. The conventional refinement approach yielded accurate values of the Flack parameter, but with standard uncertainties ranging from 0.15 to 0.77. The other methods also yielded accurate values of the Flack parameter, but with much higher precision. Absolute structure was established in all cases, even for a hydrocarbon. The procedures in which restraints are coded explicitly in terms of other structural parameters enable the Flack parameter to correlate with these other parameters, so that it is determined along with those parameters during refinement.

## Abstract 2

Differences and quotients can be defined using Friedel pairs of reflections and applied in refinement to enable absolute structure to be determined precisely even for light atom crystal structures.

## 1. Introduction   

When applied in crystallography the term *absolute structure* refers to the spatial arrangement of the atoms of a physically identified non-centrosymmetric crystal and its description by way of unit-cell dimensions, space group and representative coordinates of all atoms (Flack & Bernardinelli, 1999 ▶, 2008*b* ▶). Since inverted images of a non-centrosymmetric crystal structure are different, the question of absolute structure arises during analysis of any non-centrosymmetric crystal structure. The most important practical application of absolute structure refinement is, however, in the crystallographic determination of the absolute configuration of chiral molecules.

The fact that absolute structure can be obtained at all in a crystal structure determination is the result of resonant scattering, also known as anomalous scattering or anomalous dispersion, which introduces small differences in intensity between reflections **h** and 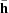 which carry the information on absolute structure. Methods for absolute structure determination most commonly used today are based on a formulation first described by Flack (1983 ▶), in which the crystal under investigation is considered to be an inversion twin in which the reference domain has the absolute structure of the current refinement model, and the other domain is inverted. Measured intensities are then modelled according to
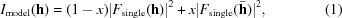
where |*F*_single_(**h**)|^2^ and |*F*_single_(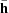)|^2^ are model quantities based on a single crystal comprised of the reference domain. The two alternative absolute structures can be refined competitively against one another by refining the twin scale factor, *x*, which in this application is referred to as the Flack parameter.

The Flack parameter has a physically meaningful value in the range of 0–1, and represents the fraction of the inverted structure present in the crystal. A value of *x* = 0 implies that none of the crystal is in the inverted form and the model has the correct absolute structure; if *x* = 1 then all of the crystal is in the inverted form. Intermediate values of *x* point to inversion twinning.

It is important to interpret the value of the Flack parameter in the context of its standard uncertainty. From a statistical point of view, a value of 0.2 (8) has such a large standard uncertainty (0.8) that one neither knows whether the crystal is twinned by inversion or not, nor whether it is inverted or not. Further analysis shows that before any conclusions regarding absolute structure can be made, the standard uncertainty of the Flack parameter should be less than 0.1, even if a material is known to be enantiopure (Flack & Bernardinelli, 2000 ▶).

The ability to achieve a low standard uncertainty for the Flack parameter depends in part on the resonant scattering effects having sufficient magnitude to lead to measurably different intensities for Friedel pairs of reflections with indices **h** and 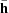. This depends on the chemical elements present in the crystal and the wavelength of the X-rays used to collect the diffraction data. The magnitude of resonant scattering effects in a given experiment can be conveniently quantified by the Friedif_stat_ parameter (Flack & Shmueli, 2007 ▶).

If Friedif_stat_ has a value of ∼ 80 or more, absolute structure determination presents little problem (Flack & Bernardinelli, 2008*a* ▶). However, resonant scattering effects for elements such as C, N and O are small for commonly available X-ray energies making it difficult to determine the Flack parameter with sufficient precision to establish absolute structure for many organic compounds. For example, the value of Friedif_stat_ for the amino acid l-alanine with Cu *K*α radiation is only 34. Accordingly, the value of the Flack parameter obtained from a conventional refinement of l-alanine was −0.04 (27). The data-set was of excellent quality, yielding low merging and refinement residuals, yet the precision of the Flack parameter is too low to enable a definitive statement to be made regarding the absolute structure (Flack & Bernardinelli, 2000 ▶).

The ability to determine absolute structure precisely also depends on low levels of random and systematic errors in intensity measurements. Analysis of non-centrosymmetric crystal structures published in *Acta Cryst. C* in 2007, 2011 and 2012 has shown that even the intensity data of structures with large Friedif_stat_ values may be entirely dominated by random uncertainties and systematic errors (Flack, 2012 ▶; Flack *et al.*, 2011 ▶).

There is a long-standing interest in finding ways to improve the precision of the Flack parameter in light-atom structures. A post-refinement Bayesian statistical procedure has been described by Hooft *et al.* (2008 ▶, 2010 ▶), which can be used either to define a probability that a refined absolute structure is correct or to obtain an estimate of the Flack parameter.^1^ Methods in which refinement weights are modified for data in proportion to their sensitivity to the Flack parameter have also been described (Bernardinelli & Flack, 1985 ▶; Parsons, Wagner *et al.*, 2012 ▶). It has further been shown that precision may be improved by the use of aspherical scattering factors (Dittrich *et al.*, 2006 ▶).

While each of the methods described has been shown to yield lower standard uncertainties on Flack parameters than conventional refinement, all are open to potential criticisms. The Hooft method, being a post-refinement method, does not formally allow *x* to correlate with other parameters during refinement, and this, theoretically at least, may compromise values of the standard uncertainty obtained (Hooft *et al.*, 2008 ▶). Reweighting methods, involving the selection of data for up-weighting, can magnify errors in the intensity measurements, so that values of *x* can be precise but inaccurate. Parsons, Wagner *et al.* (2012 ▶), for example, refer to one structure where the elimination of just two poorly measured data points shifted the Flack parameter from 0.35 (12) to 0.02 (14). Use of aspherical scattering factors yields improvements in precision, but in most of the examples tested the change was too small to enable sufficiently precise absolute structure determination for light-atom compounds (see Table 3 in Dittrich *et al.*, 2006 ▶). This said, the use of invariom models in combination with other methods, described above and herein, merits further investigation.

In this paper we will describe methods based on intensity differences and quotients that enable *x* to be refined along with all other parameters. The purpose of this paper is to demonstrate that this leads to more precise estimates of *x* than conventional refinement methods while avoiding the potential criticisms discussed above.

## 2. Definitions of intensity differences and quotients   

### 2.1. Differences   

Differences between the observed intensities of Friedel pairs of reflections
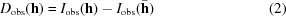
can be modelled following equation (1) with
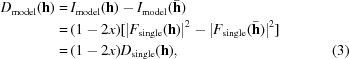
where 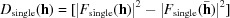. The quantities *D*, which are also referred to as Friedel or Bijvoet differences, have been used in strategies for absolute structure determination described by Hooft *et al.* (2008 ▶, 2010 ▶) and Le Page *et al.* (1990 ▶), in a procedure available in the *DIRDIF* suite of programs (Beurskens *et al.*, 1996 ▶) and in the procedure described by Thompson & Watkin (2011 ▶).

### 2.2. Quotients   

In principle, on a four-circle diffractometer equipped with a point detector it is possible to measure the intensities of reflections **h** and 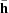 at setting angles (2θ, ω, χ and ϕ) and (−2θ, −ω, χ and ϕ) (Le Page *et al.*, 1990 ▶). The first set of setting angles is appropriate for reflection *hkl* and the second set for 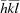. In the second set, both the incident and reflected beam directions are reversals of those of the first set. If a crystal has a centrosymmetric habit then the beam paths through the crystal of these two measurements are identical. Consequently, their absorption and extinction corrections are identical, and the quotient *I*_obs_(**h**)/*I*_obs_(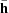) is absorption and extinction (and scale) free.

The corresponding model quotient can be written in terms of |*F*_single_(**h**)|^2^ and |*F*_single_(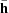)|^2^

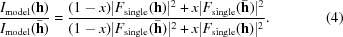
While this type of formulation has been used in the structure refinement of the kinase inhibitor roscovitine (Wang *et al.*, 2001 ▶), it is non-linear in *x*, and the standard uncertainties of quotients defined for **h** and 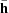 are not the same. These problems are removed, and the independence from absorption, extinction and scale maintained, by reformulating the quotients in terms of sums and differences of Friedel-pair intensities, so that the observed quotients
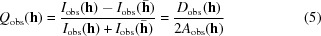
are modelled with
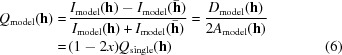
where
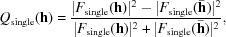


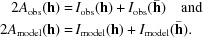



All of the test data-sets used in this study were collected using modern diffractometers equipped with area detectors. These do not in general perform reversed beam-path measurements, and so our initial contention that quotients can be measured in such a way that errors cancel does not hold.^2^ For such data we follow Parsons, Pattison & Flack (2012 ▶) in writing
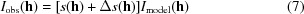


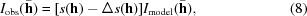
where *s*(**h**) and Δ*s*(**h**) are the average and half-difference of the systematic errors in reflections *I*_obs_(**h)** and *I*_obs_(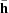) remaining after the application of a multi-scan correction. If the term 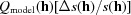 is small enough that 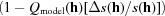 is a good approximation for 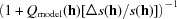, this leads to
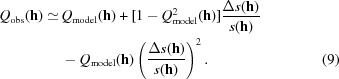
In practice |Q_model_(**h**)| is usually considerably less than 1, and so provided the difference in systematic errors in *I*_obs_(**h)** and *I*_obs_(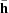) is small relative to the overall systematic error, the assumption that *Q*_model_(**h**) = *Q*_obs_(**h**) should still hold approximately.

Equation (6) can also be interpreted as being equivalent to equation (3) with an additional weighting term, 1/2*A*_model_(**h**). The incorporation of this term can be justified on the basis of the leverage analysis presented in Parsons, Wagner *et al.* (2012 ▶), in which it was shown that the data with the greatest influence on the precision of the Flack parameter were those with weak to moderate intensities. The factor 1/2*A*_model_(**h**) up-weights these data. Very weak data, which have little leverage on the Flack parameter but high values of *Q*_obs_(**h**) on account of a small value of *A*_obs_(**h**), should be omitted from an analysis based on quotients.

## 3. Experimental   

### 3.1. Data-sets   

A series of test data-sets was used in this study; selected crystal data are given in Table 1 ▶. The compounds selected for study contain no element heavier than oxygen, and all have Friedif_stat_ of 36 or less for Cu *K*α radiation. *R*-Mandelic acid and l-alanine were obtained from Sigma–Aldrich and were used as received; for other samples solution-phase optical rotation measurements or chiral separation established enantiomeric excesses of > 98%. Resonant scattering factors *f*′ and *f*′′ are independent of resolution, and the contribution of resonant relative to non-resonant scattering is therefore greatest at high values of sin θ/λ. For this reason the test data-sets were collected at low temperature. Data-sets were highly redundant, with average multiplicities of observations between 8 and 35.

Data-sets for the samples listed in Table 1 ▶ carrying the superscript A were collected with Cu *K*α radiation on an Agilent Technologies SuperNova incorporating a microsource generator. The temperature of data collection was 150 K except for *R*-mandelic acid. This material crystallizes as plates, and cooling to 150 K was found to cause strain-broadening, and so data were collected at 220 K. Processing, including integration and a multi-scan absorption correction (Blessing, 1995 ▶), was accomplished with *CrysAlis PRO* (Oxford Diffraction Ltd, 2010 ▶).

Data-sets carrying the superscript B were collected using Cu *K*α radiation at 100 K using a Bruker Microstar fine-focus rotating anode generator with a SMART 6000 CCD detector or a Bruker D8 microsource, also equipped with a SMART 6000 detector. Data were processed with *SAINT* (Bruker–Nonius, 2006 ▶) and corrected for absorption and other systematic errors using the multi-scan procedure *SADABS* (Bruker-Nonius, 2006 ▶; Sheldrick, 2008*a* ▶).

Data were merged using *SORTAV* with unit weights and robust-resistant down-weighting of outliers (Blessing, 1997 ▶).^3^

### 3.2. Absolute structure refinement in *SHELXL*2012   

Structures were solved using direct methods (*SHELXS*; Sheldrick, 2008*b* ▶) or charge flipping (*SUPERFLIP*; Palatinus & Chapuis, 2007 ▶) and refined against |*F*|^2^ in *SHELXL*2012 (beta test version 2012/9) using all data (Sheldrick, 2012 ▶). Data were merged in point groups 2, 222 or 3 for the monoclinic, orthorhombic and trigonal structures, respectively. Isopropyl groups in structures *R*- and *S*-CYCLO are disordered over two orientations (Wang *et al.*, 2001 ▶). The disorder components were restrained to have similar bond distances and angles. The water of crystallization in structure A0034a is disordered about a crystallographic twofold axis, which also induces disorder in two hydroxyl H-atom positions. All full-weight non-H atoms were refined with anisotropic displacement parameters. Full weight H atoms were refined freely; those part of disordered groups were either refined with restraints (A0034a) or placed in ideal positions (*R*- and *S*-CYCLO). The Flack parameter was refined either by full-matrix least squares (*i.e.* using the TWIN/BASF commands in the *SHELXL* .ins file) or post-refinement based on quotients defined in §2.2. The results of the first of these refinements were used to calculate the Hooft parameter *via* the BIJVOET routine in *PLATON*. In all cases a Gaussian prior was used (Hooft *et al.*, 2008 ▶; Spek, 2003 ▶).

### 3.3. Absolute structure refinement in *CRYSTALS*   

The same models as described above were refined in *CRYSTALS* (Version 14.40; Betteridge *et al.*, 2003 ▶) and the Flack parameter estimated *via* the procedure described by Thompson & Watkin (2011 ▶).

### 3.4. Absolute structure refinement in *TOPAS-Academic*   

*TOPAS-Academic*, Version 5 (Coelho, 2012 ▶), allows user-equations to be written in the form of a function, in a similar way to the definition of a function or subroutine in a programming language such as Fortran or C++ (Coelho *et al.*, 2011 ▶). These equations can then be used in restraints or to define other parameters. This option enables equation (6) to be incorporated into the refinement in the form of a set of restraints where the equation is coded in terms of atomic positional, displacement and occupancy parameters for each quotient.

The quotients *Q*_obs_(**h**) were calculated from the integrated data-sets using reflections for which both *I*_obs_(**h**) and *I*_obs_(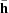) were greater than three times their respective uncertainties. This cut-off condition eliminates quotients which are large because the term *A*_obs_(**h**) is small (see §2.2). *Q*_obs_(**h**) may also be calculated using *XPREP* (Sheldrick, 2001 ▶). Outlier data for which |*D*_obs_(**h**)| was greater than twice the maximum value of |*D*_single_| were also excluded. This condition mirrors the procedure used for outlier detection in the Bijvoet routine in *PLATON* (Spek, 2003 ▶). For surviving data the standard uncertainty *u*[*Q*_obs_(**h**)] was propagated from the values of *u* [*I*_obs_(**h**)] and *u* [*I*_obs_(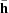)] obtained from merging
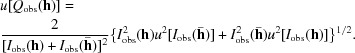



The observations used in *TOPAS* for refinement took the form of Friedel-averaged intensity data, *A*_obs_(**h**), and quotients, *Q*_obs_(**h**), the latter in the form of restraints.

The values of *A*_obs_(**h**) and their uncertainties were obtained by merging the centric and paired acentric data in the relevant Laue group (*e.g. mmm* for an orthorhombic structure). Any unpaired acentric data were omitted. The structure was first refined in *CRYSTALS* against *A*_obs_(**h**) only, and the weighting scheme optimized. This weighting scheme was then held fixed in subsequent refinement in *TOPAS*. The *A*_obs_(**h**) values were modelled both in *CRYSTALS* and *TOPAS* using a Flack parameter equal to 0.5 in order to correctly account for the averaging of Friedel-pair intensities.

The quotient data, *Q*_obs_(**h**), were modelled with equation (6) using a second Flack parameter; it is this parameter which characterizes the absolute structure. The quotient restraints were initially given a weight, *w*_restraint_(**h**) = 1/*u* ^2^ [*Q*_obs_(**h**)]. After initial cycles of refinement the values of the deviates 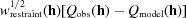 were used to calculate a reduced χ^2^ statistic. The structure was then re-refined with the quotient restraint weights scaled by 1/χ^2^. A normal probability plot (Abrahams & Keve, 1971 ▶) of deviates was also inspected to detect further outliers and for validation purposes.

The same procedure was applied to the refinement using difference restraints based on equation (3). No cut-off condition based on values of *I*_obs_(**h**)/*u*(*I*_obs_(**h**)) was applied, but outliers were detected as described above. The initial restraint weights were *w*_restraint_(**h**) = 1/*u* ^2^ [*D*_obs_(**h**)] = 1/(*u* ^2^ [*I*_obs_(**h**)] + *u* ^2^[*I*_obs_(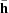)]), but these were rescaled after initial cycles of refinement, also as described above.

Clearly it is only possible to calculate *Q*_obs_(**h**) and *D*_obs_(**h**) for acentric data where both *I*_obs_(**h**) and *I*_obs_(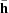) have been measured; coverage statistics along with numbers of data omitted as outliers are given in Table 2 ▶. Input files for quotient refinement of *L*-alanine^B^ in *TOPAS-Academic* are available in the supplementary material.

Programs written to calculate *Q*_obs_(**h**) and *D*_obs_(**h**), detect outliers and write files of symbolic restraints made use of subroutines available in the CrysFML Fortran library (Rodríguez-Carvajal & González Platas, 2009 ▶).

## 4. Results and discussion   

### 4.1. Conventional refinement of the Flack parameter   

The results of ‘conventional’ refinement of *x*, that is as a twin factor in full-matrix least squares, are listed in Table 1 ▶ in the column *x*(twin). They are completely consistent with the results of Flack & Bernardinelli (2008*a* ▶), which indicates that compounds with Friedif_stat_ in the range 9–36 would be expected to yield standard uncertainties of the Flack parameter of between 0.8 and 0.2. None of the conventional refinements yields a Flack parameter with sufficient precision to enable a conclusion to be made about the absolute structures of the crystals being studied.

It is, however, remarkable that the values of the Flack parameter in Table 1 ▶ cluster around zero much more tightly than would be anticipated on the basis of their high standard uncertainties. The reduced χ^2^ calculated from the data in the *x*(twin) column (assuming the true value of *x* is zero in each case) is only 0.031, suggesting that the uncertainties are overestimated by a factor of 5.5, meaning that more information about absolute structure is present in the data than implied by the uncertainties calculated by least squares. While this finding runs counter to the general *underestimation* of standard uncertainties in crystallographic least squares (Hamilton & Abrahams, 1970 ▶), it is consistent with Thompson & Watkin’s (2011 ▶) histogram of Flack parameters obtained from refinements of 150 structures with Friedif_stat_ in the range 3.4–10.8. The histogram was centred at zero; had the data been devoid of absolute structure information, the histogram should have been centred about 0.5.

It appears that conventional refinement of *x* yields rather pessimistic uncertainty estimates. Although the data quoted in Table 1 ▶ were derived using *SHELXL*2012, similar results were obtained with *CRYSTALS* and *TOPAS*. The methods published elsewhere by Hooft *et al.* (2008 ▶, 2010 ▶), Thompson & Watkin (2011 ▶) and Parsons, Wagner *et al.* (2012 ▶) and those described below attempt to obtain more realistic uncertainty estimates.

### 4.2. Post-refinement estimation of the Flack parameter using quotients   

Values of *Q*_obs_(**h**) [equation (5)] can be calculated using the observed intensity data, while values of 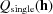 [equation (6)] can be calculated from the refinement model. A ‘*Q* plot’ of *Q*_obs_(**h**) against 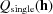 should be a straight line passing through the origin with a gradient of (1 − 2*x*). Similarly, a ‘*D* plot’ of *D*_obs_(**h**) [equation (2)] against 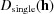 [equation (3)] should also be linear with a gradient of (1 − 2*x*).

**Figure 1:**
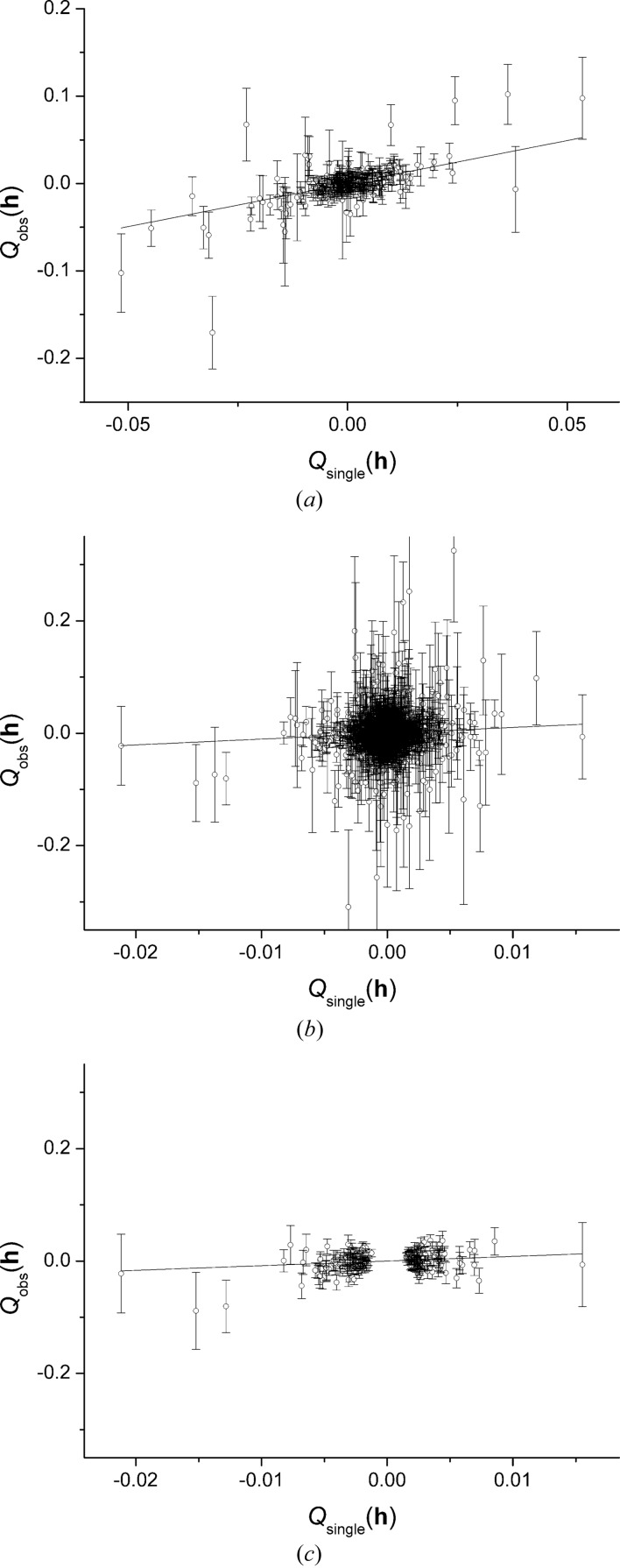
Plot of *Q*_obs_(**h**) against *Q*_single_(**h**) [as defined in equations (5) and (6)] for (*a*) l-alanine and (*b*) cholestane. (*c*) as (*b*), but only the top 200 most influential points are shown; the axes are chosen to be the same as (*b*).

The method can be illustrated with reference to the data collected for l-alanine. The gradient of the weighted least-squares best straight line in the *Q* plot shown in Fig. 1 ▶(*a*) is 0.984 (68), which yields a value of the Flack parameter of 0.01 (3). This estimate is very much more precise than that obtained with the same data in a conventional refinement. Some of the error bars in Fig. 1 ▶ are huge, but these points have little influence on the (weighted) fit. Much more important is the very clear unit slope in the bulk of the data points.

As the effects of resonant scattering become smaller, the trend in *Q*-plots becomes harder to discern by eye, as illustrated in the plot for cholestane (Friedif_stat_ = 9) in Fig. 1 ▶(*b*). The least-squares fit for the data shown nevertheless yields a Flack parameter of −0.02 (12). Part of the problem in Fig. 1 ▶(*b*) is that the majority of points have little influence on the fit. Fig. 1 ▶(*c*) shows the 200 most influential points in Fig. 1 ▶(*b*) (as measured by their leverages; Parsons, Wagner *et al.*, 2012 ▶; Prince, 2004 ▶; Merli *et al.*, 2000 ▶). The noticeable gap in the middle of the plot occurs because for a one-parameter linear fit the leverages are proportional to 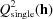; other missing points in Fig. 1 ▶(*c*) have a low leverage on account of their high error bars. Although not exactly obvious, the trend in Fig. 1 ▶(*c*) is a little clearer than it is in Fig. 1 ▶(*b*). More significantly the value of *x* obtained for these points is 0.09 (15): even though we are only working with ∼ 5% of the data, the precision is only marginally affected.

This linear *Q*-fitting method has been incorporated into *SHELXL*2012, and the results for the other compounds studied are the top values listed in the column labelled ‘*x*(quotient)’ in Table 1 ▶. Also included in Table 1 ▶ [in column *y*(Hooft)] are the estimates of Hooft’s Bayesian method. The results of the two procedures are in excellent agreement, both showing consistently higher precision than conventional methods [*x*(twin)].

### 4.3. Estimation of the Flack parameter using quotient data   

Although the method described in §4.2 yields precise and accurate values of the Flack parameter, like other post-refinement methods, it has the disadvantage that *x* is not allowed to correlate with other parameters. A way around this difficulty is to incorporate equation (6) into the structure refinement (Murphy *et al.*, 2010 ▶; Parsons & Flack, 2004 ▶; Wang *et al.*, 2001 ▶). Values of *Q*_obs_(**h**) defined in equation (5) are calculated from the intensity data, as before, but facilities available in *TOPAS* enable *Q*_single_(**h**) to be written in terms of the parameters of the refinement model (atomic coordinates, displacement parameters and occupancies) and built into the refinement as an equation of restraint; an example is provided in the supplementary material. The Flack parameter can now correlate with other parameters during refinement.

A quotient can be defined for each Friedel pair of intensities in the data-set so that the observations in the refinement now take the form of Friedel-averaged intensity measurements, *A*_obs_(**h**), and the quotients, *Q*_obs_(**h**). The values of *A*_obs_(**h**) are obtained by merging the centric and paired acentric data in the centrosymmetric Laue group, and all absolute structure information is contained in the quotients. The number of quotients used in the test examples lay between a few hundred to several thousand depending on the size of the structure (details are in Table 2 ▶).

The bottom values listed in the *x*(quotient) column of Table 1 ▶ show the results. They are very similar to those obtained with the post-refinement procedure described in §4.2, and much more precise than those obtained with the conventional refinement technique. Acceptable precision has been obtained for data-sets with Friedif_stat_ as low as 12.

**Figure 2:**
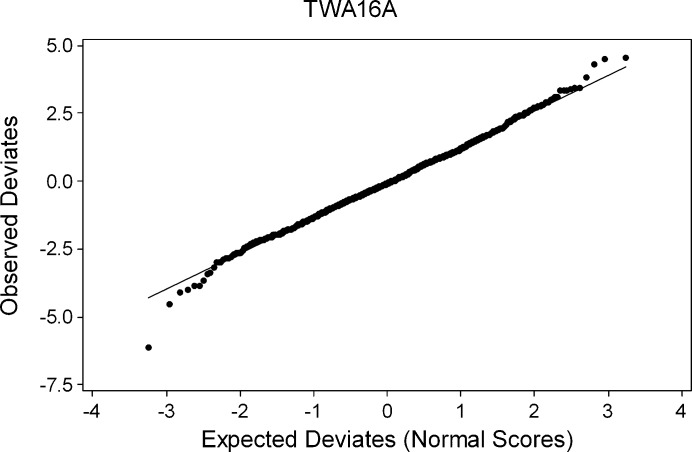
Outlier detection. Normal probability plot calculated using observed quotient restraint deviates 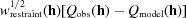 for TWA16a. The extreme point at the bottom left was omitted as an outlier. This plot was calculated prior to rescaling of weights.

The Flack parameter is sensitive to outliers in the data, and it is important that these are detected and eliminated. The sensitivity to outliers can be illustrated using the data-set TWA16a. A refinement performed with no outlier elimination at all yielded a Flack parameter equal to 0.18 (8). A normal probability plot calculated for this refinement (Fig. 2 ▶) was linear, but with one point (at the bottom left in Fig. 2 ▶) deviating substantially from the straight line. Elimination of this one observation changed the value of *x* to 0.08 (8).

Normal probability plots are a powerful means for detecting outliers, although in this work we follow Spek’s procedure in *PLATON* in eliminating Friedel pairs with |*D*_obs_(**h**)| more than twice the maximum calculated absolute difference for the entire data-set. This is a more objective procedure, although it means that more data are omitted the lower the value of Friedif_stat_. The numbers of outliers omitted are given in Table 2 ▶.

### 4.4. Estimation of the Flack parameter using difference data   

An alternative procedure is to base the restraints on differences rather than quotients. The target value for each restraint is *D*_obs_(**h**) as defined in equation (2). The model value *D*_model_(**h**) is defined in equation (3) and written in terms of the refinement parameters. The procedure was otherwise identical to that described in §4.3.

A similar procedure is available in *CRYSTALS*. Here refinement is carried out against |*F*_obs_(**h**)|^2^ data merged in the appropriate crystal (as opposed to Laue) point group and *D*_model_(**h**) is a numerical value calculated from the current model (see Fig. 2 ▶ in Thompson & Watkin, 2011 ▶).

The results of the two procedures are listed in the *x*(difference) column of Table 1 ▶; the top value is obtained from the *CRYSTALS* procedure, the bottom *via* explicit restraints. The results are in agreement with each other and those obtained for quotients.

### 4.5. The extent of error cancellation on taking quotients   

The differences defined in equations (2) and (3) are used in the Bayesian and numerical restraint methods for estimation of the Hooft or Flack parameters available in *PLATON* and *CRYSTALS*. Our original idea of basing absolute structure analysis on quotients was conceived about a decade ago, when four-circle instruments with point detectors were still in common use. The cancellation of absorption and extinction errors which occurs on taking intensity quotients using reverse-beam measurements of Friedel pair intensities cannot in general be achieved with modern area-detector instruments (but see footnote 2, §2.2). The analysis presented in §2 suggests that some approximate cancellation of errors occurs provided the difference in systematic errors in *I*_obs_(**h)** and *I*_obs_(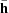) is small relative to the overall systematic error.

One means for assessing whether error cancellation is achieved in practice with area-detector data is to compare *R* factors based on the observed and model values of quotients or differences [equation (10)]. If errors are really cancelled *R*(*Q*) should be systematically lower than *R*(*D*).
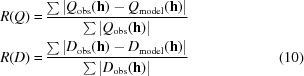
Values of *R*(*Q*) and *R*(*D*) are listed for each data-set in Table 3 ▶. The differences are mostly quite marginal, a finding consistent with a similar listing in Table 10 of Parsons, Pattison & Flack (2012 ▶).

A second method for assessing the presence of systematic errors is to examine normal probability plots based on the weighted residuals 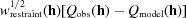 and 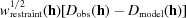 for the quotient and difference refinements, respectively. Systematic errors shift the intercept of the plot away from the origin and cause the plot itself to deviate from linearity. The intercepts, gradients and Pearson correlation coefficients (*r* ^2^) of normal probability plots for quotient and difference refinements are also listed in Table 3 ▶. There is generally rather little systematic difference between the intercepts calculated for quotients or differences, all falling very close to the origin. There is no systematic difference between the correlation coefficients for the quotient and difference plots. The gradients are all near the ideal value of unity, but this is a consequence of the weight-scaling described in §3.4.

The *R*-factor and normal probability calculations indicate that cancellation of systematic errors can occur on taking quotients of intensities collected with area detectors, but the improvement, if present, is usually small and the results of using either method essentially the same. This is possibly because the assumptions about relative systematic errors referred to above are not met or because absorption and extinction are not the principal systematic errors present.

The linearity and small intercepts of the normal probability plots listed in Table 3 ▶ indicate that the weights applied to the quotients and differences reflect the uncertainties in the data. The values of reduced χ^2^ listed in Table 1 ▶ are near unity for both quotient and difference-based methods, indicating that the magnitudes of the standard uncertainties are realistic. Taken with the accuracy of the values of the Flack parameters listed in Table 1 ▶ this shows that quotient and difference methods are both appropriate for absolute structure determination.

### 4.6. Leverage analysis   

Some insight into why the methods presented increase the precision in *x* can be gained by considering the relative influences of the observations *A*_obs_(**h**) and *Q*_obs_(**h**) or *D*_obs_(**h**) on the structural and Flack parameters. Leverage analysis was carried out on the refinement of l-alanine^B^ using *CRYSTALS* (Parsons, Wagner *et al.*, 2012 ▶).

**Figure 3:**
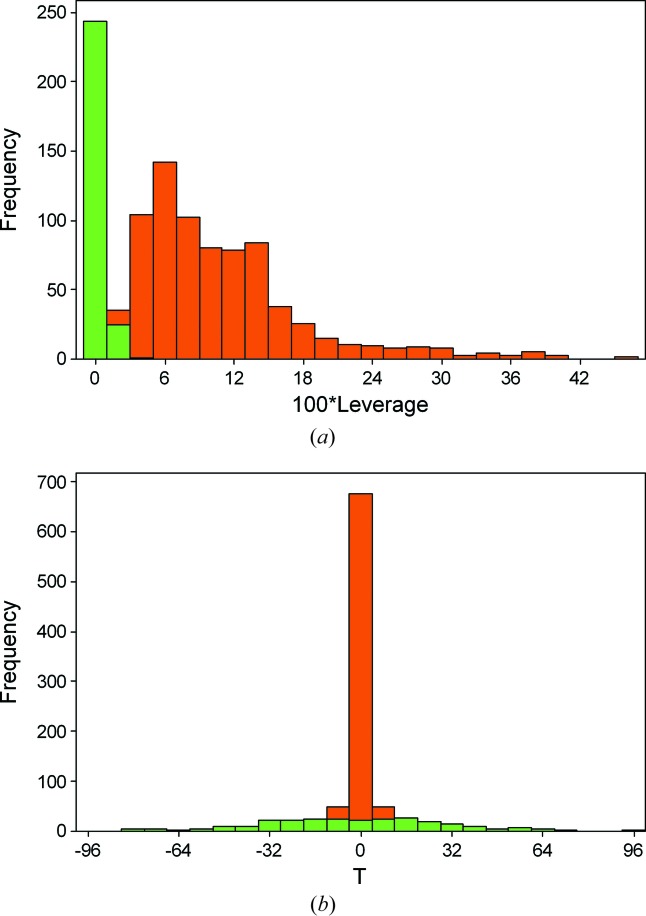
Leverage analysis for l-alanine^B^. (*a*) Leverages of |*F*_obs_(**h**)|^2^ and difference data, *D*_obs_(**h**). (*b*) Relative influences of |*F*_obs_(**h**)|^2^ and *D*_obs_(**h**) on the precision of the Flack parameter (as expressed by the quantity *T*). In each case |*F*_obs_(**h**)|^2^ data are shown in orange and *D*_obs_(**h**) data in green.

Fig. 3 ▶(*a*) is a histogram of leverages for the |*F*_obs_(**h**)|^2^ (orange) and the *D*_obs_(**h**) (green) data. The *D*_obs_(**h**) leverages cluster near zero showing that they have rather little effect on the overall data-fitting. The insensitivity of the structural parameters to the Friedel difference intensities reflects a similar finding described in §4.1 of Flack *et al.* (2011 ▶).

Fig. 3 ▶(*b*) shows a histogram of the quantity *T*, which measures the influence of observations on the precision of the Flack parameter (David, 2004 ▶; David *et al.*, 1993 ▶; Parsons, Wagner *et al.*, 2012 ▶; Prince, 2004 ▶). Here the situation seen in the leverage plot is reversed, the orange |*F*_obs_(**h**)|^2^ data cluster about zero, whereas the green *D*_obs_(**h**) data span the range ±100.

The improvement in the precision of the Flack parameter which is gained by using differences or quotients is the result of transforming the observations into one set [*A*_obs_(**h**)] which is sensitive to the structure but independent of the Flack parameter, and another [*D*_obs_(**h**) or *Q*_obs_(**h**)] which is sensitive to the Flack parameter, but very insensitive to the atomic parameters. The transformation means that correlation between the Flack parameter and the other refined parameters is essentially absent, and this explains why the results of the post-refinement methods are so similar to those obtained with the method outlined above.

## 5. Concluding remarks   

Absolute structure refinements have been carried out for a series of 23 light-atom crystal structures with Friedif_stat_ values for Cu *K*α radiation of between 9 and 36. Accurate values of the Flack parameter and its standard uncertainty were obtained, but with a precision higher and more realistic than conventional refinement. The results of the methods implemented in *SHELXL*2012, *CRYSTALS* and *PLATON* are essentially the same as those obtained when quotients or differences are explicitly coded into the refinement in *TOPAS*. We conclude that the potential problems discussed in §1 associated with a lack of correlation between the Flack parameter and the other structural parameters are not significant in absolute structure determinations of light-atom compounds. This justifies the use of post-refinement algorithms for absolute structure determination provided a complete set of Friedel pair intensity measurements is available.

On the basis of the results presented in the current paper, and those cited herein, it is possible to provide an outline of the treatment of diffraction data which leads to a reliable value of the Flack parameter with as low and realistic standard uncertainty as possible. The steps are described below in terms of intensity differences (*D*), although they could equally well be applied to quotients (*Q*)

(i) The reflection data-set is separated into three disjoint classes consisting of centric reflections (class *c*), pairs of Friedel opposites of acentric reflections (class *ap*) and unpaired acentric reflections (class *au*). If the data-set contains an unacceptably large proportion of unpaired acentric reflections, the diffraction data should be recollected with a revised collection strategy. The data in class *au* are omitted from further analysis.
(ii) The intensities of the pairs of acentric reflections (class *ap*), are transformed into averages, *A*_obs_(**h**), and differences, *D*_obs_(**h**).
(iii) Least-squares refinement of the structural parameters is undertaken using as data the centric reflections (class *c*) and the average intensities of the paired Friedel opposites [*A*_obs_(**h**)]. This refinement corresponds to a crystal twinned by inversion in a proportion of 50:50, so a value of the Flack parameter fixed at 0.5 should be used. The resulting atomic parameters are unbiased by the effects of resonant scattering and inversion twinning.
(iv) Using the atomic parameters obtained from stage (iii)[Other l1li3] and a Flack parameter of 0.0, structure-factor amplitudes are calculated for the pairs of Friedel opposites in class *ap*. This leads to model values *D*_single_(**h**) corresponding to a single crystal untwinned by inversion.
(v) From a plot of *D*_obs_(**h**) against *D*_single_(**h**) one has a powerful tool for validating the absolute-structure determination and obtaining a value of the Flack parameter from a least-squares fit to a straight line passing through the origin. Critical to the success of this fit is the choice of a suitable weighting scheme and the elimination of outliers. The texts of the current paper, and those cited, contain the corresponding information.

The transformation of the data described in (ii)[Other l1li2] yields one set of observations [the centric data and *A*_obs_(**h**)] which is sensitive to the structure but independent of the Flack parameter, and another [*D*_obs_(**h**) or *Q*_obs_(**h**)] which is sensitive to the Flack parameter, but highly insensitive to the atomic parameters. The agreement between *A*_obs_(**h**) and *A*_model_(**h**) is usually much better than between *D*_obs_(**h**) and *D*_model_(**h**) or their equivalents based on quotients (*e.g.* Flack *et al.*, 2011 ▶; Parsons, Pattison *et al.*, 2012 ▶); one advantage of the transformation of data into *A* and *D* (or *Q*) is that different schemes for the selection of outliers and weights can be applied to each. A conventional refinement is compatible only with a single ‘one-size-fits-all’ weighting scheme. Use of the transformed data also removes correlation between the Flack parameter and the other structural parameters.

All data in this work were collected with Cu *K*α radiation, although there is no reason why the methods described could not be applied to data from more than one source, for example, Cu *K*α radiation for the Friedel-averaged intensity data *A*_obs_(**h**) and Cr *K*α radiation for the difference or quotient data *D*_obs_(**h**) or *Q*_obs_(**h**). This will be investigated in due course.

## Supplementary Material

Contents of supplementary data. DOI: 10.1107/S2052519213010014/gp5062sup1.pdf

Supporting information file. DOI: 10.1107/S2052519213010014/gp5062sup2.zip

Supporting information file. DOI: 10.1107/S2052519213010014/gp5062sup3.zip

Supporting information file. DOI: 10.1107/S2052519213010014/gp5062sup4.zip

## Figures and Tables

**Table 1:** Results of absolute structure refinements using the methods outlined in the text *R*_1_[|*F*| > 4(|*F*|)] is the unweighted *R* factor based on |*F*| for the intensity data only. The *x*(twin) column contains values of the Flack parameter (*x*) calculated in the ‘conventional’ manner against intensity data merged in the relevant non-centrosymmetric point group (*SHELXL*2012); the output of these refinements was used to calculate *y*(Hooft) using Bayesian methods (*PLATON*) and the value of *R*_1_[|*F*| > 4(|*F*|)]. In the *x*(quotient) column the top figure refers to post-refinement calculation of *x* as described in 4.2 (*SHELXL*2012) and 4.1; the bottom figure refers to the refinement performed as in 4.3 with the intensity quotients applied as restraints during refinement (*TOPAS*). The top figure in the *x*(difference) column was obtained from the numerical restraints method implemented in *CRYSTALS* and the bottom figure to the fully coded difference restraints (4.4) in *TOPAS*. The superscripts A and B in the ‘Structure code’ column refer to data collected with Agilent or Bruker instruments, respectively. Samples TWA16a and TWA16b are polymorphs; TWA17c is a recollection of TWA17a with a different sample; *R*- and *S*-CYCLO, FYO11 and FYO12, and TWA17 and TWA20 are enantiomers.

Structure code	Chemical formula	Friedif_stat_	Space group	Redundancy	*R*_1_[|*F*| > 4(|*F*|)]	*x* (twin)	*y* (Hooft)	*x* (quotient)	*x* (difference)
*R*-Mandelic acid^A^	C_8_H_8_O_3_	36	*P*2_1_	11	0.0511	0.12 (46)	0.03 (6)	0.00 (11)	0.04 (5)
								0.01 (9)	0.00 (9)
L-Alanine^B^	C_3_H_7_NO_2_	34	*P*2_1_2_1_2_1_	25	0.0219	0.04 (27)	0.01 (4)	0.01 (4)	0.04 (3)
								0.01 (3)	0.01 (3)
L-Alanine^A^	C_3_H_7_NO_2_	34	*P*2_1_2_1_2_1_	15	0.0181	0.06 (26)	0.06 (5)	0.05 (4)	0.04 (3)
								0.08 (4)	0.07 (4)
Glutamine^B^	C_5_H_8_N_2_O_2_	33	*P*2_1_2_1_2_1_	28	0.0248	0.09 (25)	0.07 (3)	0.07 (3)	0.04 (2)
								0.09 (3)	0.07 (3)
GKO02^B^	C_25_H_31_NO_5_	32	*P*2_1_2_1_2_1_	15	0.0247	0.01 (15)	0.03 (3)	0.02 (3)	0.02 (2)
								0.03 (3)	0.02 (3)
A0030a^B^	C_21_H_26_N_2_O_3_	29	*P*2_1_2_1_2_1_	11	0.0263	0.10 (21)	0.07 (5)	0.07 (6)	0.05 (3)
								0.07 (5)	0.07 (5)
A0034a^B^	C_18_H_25_O_2.5_	29	*P*2_1_2_1_2	11	0.0274	0.00 (21)	0.02 (3)	0.02 (3)	0.06 (2)
								0.02 (2)	0.02 (2)
A0034b^B^	C_18_H_25_O_2.5_	29	*P*2_1_2_1_2	35	0.0268	0.01 (22)	0.02 (3)	0.02 (3)	0.01 (3)
								0.02 (2)	0.02 (2)
LRE01a^B^	C_14_H_19_NO	24	*P*2_1_2_1_2_1_	22	0.0278	0.01 (33)	0.03 (5)	0.04 (6)	0.04 (3)
								0.02 (5)	0.03 (5)
TWA18a^B^	C_16_H_20_N_2_O	23	*P*2_1_2_1_2_1_	17	0.0253	0.04 (26)	0.04 (3)	0.07 (3)	0.00 (2)
								0.04 (3)	0.04 (3)
*R*-CYCLO^A^	C_19_H_26_N_6_O	21	*P*2_1_2_1_2_1_	14	0.0425	0.02 (27)	0.02 (4)	0.00 (4)	0.02 (4)
								0.02 (4)	0.02 (4)
*S*-CYCLO^A^	C_19_H_26_N_6_O	21	*P*2_1_2_1_2_1_	16	0.0409	0.03 (20)	0.04 (3)	0.04 (3)	0.01 (3)
								0.04 (2)	0.02 (4)
TWA21a^B^	C_21_H_29_N_3_	14	*P*2_1_2_1_2_1_	17	0.0248	0.00 (40)	0.00 (4)	0.01 (4)	0.05 (4)
								0.00 (3)	0.00 (3)
TWA20c^B^	C_19_H_19_N_2_	12	*P*3_2_	26	0.0231	0.02 (46)	0.01 (5)	0.01 (6)	0.00 (4)
								0.00 (5)	0.01 (5)
TWA16a^B^	C_16_H_18_N_2_	13	*P*3_2_	13	0.0283	0.00 (69)	0.02 (7)	0.18 (8)	0.04 (5)
								0.14 (8)	0.05 (7)
TWA16b^B^	C_16_H_18_N_2_	13	*P*2_1_	8	0.0286	0.02 (37)	0.05 (6)	0.06 (6)	0.05 (5)
								0.07 (6)	0.06 (6)
TWA17a^B^	C_19_H_18_N_2_	12	*P*3_1_	9	0.0300	0.00 (60)	0.06 (8)	0.04 (9)	0.06 (7)
								0.04 (8)	0.06 (9)
TWA17c^B^	C_19_H_18_N_2_	12	*P*3_1_	15	0.0319	0.00 (63)	0.04 (5)	0.05 (7)	0.12 (11)
								0.10 (7)	0.02 (7)
TWA22a^B^	C_21_H_22_N_2_	12	*P*2_1_2_1_2_1_	11	0.0262	0.01 (41)	0.04 (7)	0.06 (6)	0.00 (6)
								0.05 (6)	0.05 (6)
FYO12d^B^	C_21_H_22_N_2_	12	*P*2_1_2_1_2_1_	35	0.0246	0.07 (53)	0.04 (9)	0.09 (9)	0.05 (6)
								0.04 (8)	0.04 (8)
FYO12e^B^	C_21_H_22_N_2_	12	*P*2_1_2_1_2_1_	35	0.0252	0.17 (54)	0.01 (8)	0.04 (9)	0.10 (6)
								0.01 (8)	0.02 (8)
FYO11d^B^	C_21_H_22_N_2_	12	*P*2_1_2_1_2_1_	18	0.0257	0.08 (53)	0.03 (6)	0.03 (5)	0.04 (5)
								0.03 (5)	0.02 (5)
Cholestane^B^	C_27_H_48_	9	*P*2_1_	18	0.0288	0.01 (77)	0.04 (9)	0.01 (13)	0.02 (8)
								0.00 (11)	0.03 (11)
Reduced ^2^						0.03	0.83	1.22	1.47
								1.47	0.86

**Table 2:** Data-set statistics The columns contain the compound identifier, the total number of data measured, the number of centric data, the number of paired acentric data used for generating difference and quotient restraints, the number of unpaired acentric data, the number of outlying pairs for which |*D*_obs_(**h**)| > 2|*D*_single, max_|, and the number of difference and quotient restraints used. The figures in brackets in the last two columns are the number of outliers omitted after normal probability plot analysis.

Code	Measured	Centric	Paired acentric	Unpaired acentric	Outlying pairs	Differences	Quotients
*R*-Mandelic acid^A^	2858	298	2546	8	3	1273	1140
L-Alanine^B^	776	189	570	5	6	285	284
L-Alanine^A^	740	184	540	0	8	270	270
Glutamine^B^	1177	265	898	2	6	449	449
GKO^B^	4073	588	3450	1	17	1725	1685
A0030a^B^	3333	518	2770	3	21	1385	1331
A0034a^B^	2757	470	2256	3	14	1128	1127
A0034b^B^	2673	463	2202	0	4	1101	1099
LRE01a^B^	2261	418	1800	3	20	900	894
TWA18a^B^	2488	407	2046	1	17	1023	1021
*R*-CYCLO^A^	7983	921	7008	12	21	3504	3454
*S*-CYCLO^A^	8240	939	7242	9	25	3620 [1]	3606 [1]
TWA21a^B^	3071	471	2520	6	37	1260	1258
TWA20c^B^	2545	0	2476	1	34	1238	1238
TWA16a^B^	2167	0	2082	73	6	1041	1036 [5]
TWA16b^B^	4711	220	4034	29	214	2017	1984
TWA17a^B^	2657	0	2580	3	37	1290	1286
TWA17c^B^	2657	0	2572	1	42	1286	1283 [3]
TWA22a^B^	2920	508	2344	4	32	1172	1168
FYO12d^B^	2901	463	2372	0	33	1186	1158
FYO12e^B^	2894	458	2380	4	26	1190	1167
FYO11d^B^	2887	453	2366	2	33	1183	1173
Cholestane^B^	8266	478	7120	2	333	3560	3546

**Table 3:** Fitting statistics for the refinements based on quotients and differences In the ‘Quotient restraints’ columns, the intercept, gradient and Pearson *r* ^2^ refer to normal probability plots calculated using weighted residuals for the restraints, 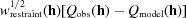. Equivalent formulae were used for the ‘Difference restraints’ columns. *R*(*Q*) and *R*(*D*) are defined in equation (10).

	Quotient restraints				Difference restraints			
Code	Intercept	Gradient	*r* ^2^	*R*(*Q*)	Intercept	Gradient	*r* ^2^	*R*(*D*)
*R*-Mandelic acid^A^	0.0461	0.993	0.986	1.003	0.0501	0.992	0.985	0.982
L-Alanine^B^	0.0127	0.995	0.982	0.776	0.0118	0.994	0.980	0.825
L-Alanine^A^	0.0503	1.002	0.997	0.892	0.0471	1.002	0.997	0.907
Glutamine^B^	0.0158	1.002	0.998	0.913	0.0219	1.002	0.998	0.904
GKO^B^	0.0019	1.000	0.998	0.925	0.0072	1.000	0.998	0.951
A0030a^B^	0.0517	0.998	0.997	0.965	0.0525	0.998	0.997	0.964
A0034a^B^	0.0036	1.000	0.997	0.873	0.0070	1.000	0.997	0.923
A0034b^B^	0.0547	0.999	0.998	0.832	0.0555	0.999	0.998	0.878
LRE01a^B^	0.1018	0.995	0.996	0.959	0.1057	0.994	0.996	0.931
TWA18a^B^	0.0175	0.999	0.996	0.886	0.0166	0.999	0.996	0.921
*R*-CYCLO^A^	0.0300	0.999	0.997	0.979	0.0269	0.999	0.998	0.981
*S*-CYCLO^A^	0.0017	1.000	0.999	0.944	0.0019	1.000	0.999	0.967
TWA21a^B^	0.0015	0.999	0.996	0.916	0.0002	1.000	0.996	0.962
TWA20c^B^	0.0138	1.000	0.998	0.961	0.0153	1.000	0.998	0.990
TWA16a^B^	0.0016	1.000	0.996	1.001	0.0030	1.000	0.993	0.998
TWA16b^B^	0.0106	0.999	0.997	0.991	0.0027	0.998	0.994	0.987
TWA17a^B^	0.0078	0.999	0.996	0.981	0.0079	0.999	0.996	0.990
TWA17c^B^	0.0159	1.000	0.998	0.990	0.0132	1.000	0.997	0.984
TWA22a^B^	0.0456	0.997	0.994	0.986	0.0440	0.997	0.994	0.984
FYO12d^B^	0.0697	0.997	0.995	0.998	0.0704	0.996	0.995	0.997
FYO12e^B^	0.0042	0.998	0.993	0.994	0.0011	0.997	0.993	0.998
FYO11d^B^	0.0196	0.998	0.994	0.964	0.0241	0.998	0.994	0.988
Cholestane^B^	0.0046	1.000	0.998	0.996	0.0048	1.000	0.998	0.999

## Footnotes

1 The Flack parameter estimated in this way is referred to as the Hooft parameter and given the symbol *y*.

2 A referee to this paper pointed out that reverse-beam measurements can be made for low-angle reflections on a four-circle diffractometer with an area detector by measuring 360° ϕ-scans at χ = +90 and −90°. In this case Friedel pairs fall on the same pixels of the detector eliminating another source of possible systematic error. The use of data collected in this way for measuring *Q*_obs_(**h**) is a very interesting avenue for further work.

3 Supplementary data for this paper are available from the IUCr electronic archives (Reference: GP5062). Services for accessing these data are described at the back of the journal.
