# Supplementary material for: Use of intensity quotients and differences in absolute structure refinement
Source: Acta Crystallogr B Struct Sci Cryst Eng Mater. 2013 May 17;69(Pt 3):249–59. doi: 10.1107/S2052519213010014 (PMC3661305; doi:10.1107/S2052519213010014)
Supplement: Supplementary file 1 [file b-69-00249-sup1.pdf]

Use of intensity quotients and differences in absolute structure refinement

Simon Parsons,[1]* Howard D. Flack[2] and Trixie Wagner[3]

[1] *School of Chemistry and Centre for Science at Extreme Conditions, The University of Edinburgh, King's Buildings, West Mains Road, Edinburgh, Scotland. EH9 3JJ*
[2] *Chimie minérale, analytique et appliquée, University of Geneva, Geneva, Switzerland.*
[3] *Novartis Institutes for BioMedical Research, 4002 Basel, Switzerland.*

Contents of Supplementary Material

1. cifs.zip
This compressed file contains cifs for all data sets in Table 1. The cifs contain final Shelxl-2012 .ins and unmerged .hkl files under data items _shelx_res_file and _shelx_hkl_file, respectively.

Intensity data for all refinements in Table 1 were merged in SORTAV so that the same merged data were used in all programs. Use of data merged with the native Shelxl-2012 merging routine will give slightly different Flack parameters to those listed in Table 1.

2. fcf.zip
This compressed file contains the fcf files corresponding to the cifs in cif.zip.

3. TA_alanine_B.zip
This compressed file contains files required for a refinement of alanine in TOPASAcademic version 5 using quotient restraints, as described in Section 3.4 of the paper. *cif2ta.inp* contains the model and the restraint data; the function QUOT used in the restraints is defined in *xtopas.dat*. The data items in *weighted.hkl* are *h*, *k*, *l*, F^2_{obs}(*hkl*) and weight(*hkl*). *cif2ta.out* is the output file after refinement.
